# *Kir6.2*-deficient mice develop somatosensory dysfunction and axonal loss in the peripheral nerves

**DOI:** 10.1016/j.isci.2021.103609

**Published:** 2021-12-11

**Authors:** Hiromi Nakai-Shimoda, Tatsuhito Himeno, Tetsuji Okawa, Emiri Miura-Yura, Sachiko Sasajima, Makoto Kato, Yuichiro Yamada, Yoshiaki Morishita, Shin Tsunekawa, Yoshiro Kato, Yusuke Seino, Rieko Inoue, Masaki Kondo, Susumu Seino, Keiko Naruse, Koichi Kato, Hiroki Mizukami, Jiro Nakamura, Hideki Kamiya

**Affiliations:** 1 Division of Diabetes, Department of Internal Medicine, Aichi Medical University School of Medicine, Nagakute 480-1185, Japan; 2 Department of Innovative Diabetes Therapy, Aichi Medical University School of Medicine, Nagakute 480-1185, Japan; 3 Department of Endocrinology, Gifu Prefectural Tajimi Hospital, Tajimi 507-8522, Japan; 4 Division of Endocrinology and Metabolism, Department of Internal Medicine, Fujita Health University School of Medicine, Toyoake 470-1192, Aichi, Japan; 5 Division of Molecular and Metabolic Medicine, Department of Physiology and Cell Biology, Kobe University Graduate School of Medicine, Kobe 650-0047, Japan; 6 Department of Internal Medicine, Aichi Gakuin University School of Dentistry, Nagoya 464-0821, Japan; 7 Department of Medicine, Aichi Gakuin University School of Pharmacy, Nagoya 464-8650, Japan; 8 Department of Pathology and Molecular Medicine, Hirosaki University Graduate School of Medicine, Hirosaki 036-8562, Japan

**Keywords:** Molecular neuroscience, Cellular neuroscience, Sensory neuroscience

## Abstract

Glucose-responsive ATP-sensitive potassium channels (K_ATP_) are expressed in a variety of tissues including nervous systems. The depolarization of the membrane potential induced by glucose may lead to hyperexcitability of neurons and induce excitotoxicity. However, the roles of K_ATP_ in the peripheral nervous system (PNS) are poorly understood. Here, we determine the roles of K_ATP_ in the PNS using K_ATP_-deficient (*Kir6.2*-deficient) mice. We demonstrate that neurite outgrowth of dorsal root ganglion (DRG) neurons was reduced by channel closers sulfonylureas. However, a channel opener diazoxide elongated the neurite. K_ATP_ subunits were expressed in mouse DRG, and expression of certain subunits including Kir6.2 was increased in diabetic mice. In *Kir6.2*-deficient mice, the current perception threshold, thermal perception threshold, and sensory nerve conduction velocity were impaired. Electron microscopy revealed a reduction of unmyelinated and small myelinated fibers in the sural nerves. In conclusion, K_ATP_ may contribute to the development of peripheral neuropathy.

## Highlights

• Transcript levels of K_ATP_ channels in DRG neurons increase in diabetic mice
• K_ATP_ channel inhibition shortens but its activation elongates neurites of DRG neurons
• *Kir6.2*^−/−^ mice present the dysfunction in the peripheral nerves
• *Kir6.2*^−/−^ mice present ultrastructural abnormalities in peripheral sensory nerves

## Graphical abstract

**Figure:**
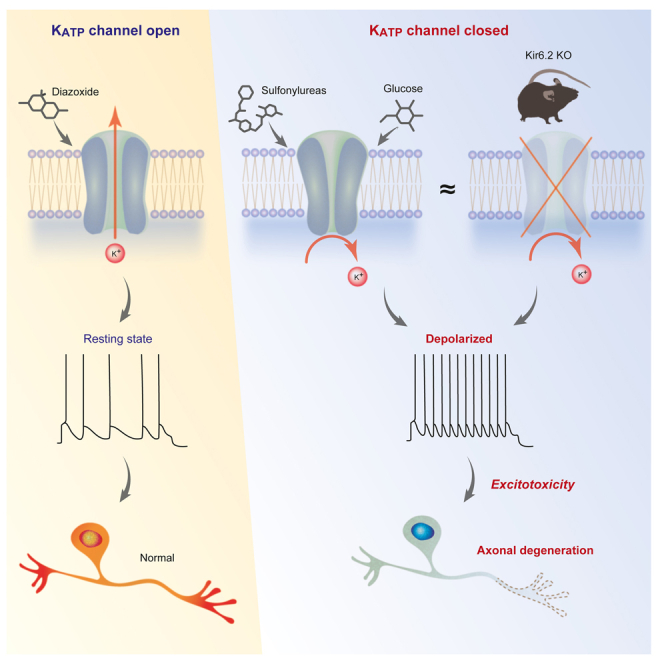

## Introduction

Among peripheral neuropathies, diabetic polyneuropathy (DPN) is the most frequent. DPN, whose prevalence in diabetic patients is up to 50% or more, is one of the most common diabetic complications and causes non-traumatic amputations of lower limbs (Nomura et al., 2018; Singleton and Smith, 2012). Although anti-hyperglycemic therapies, including insulin therapy and other glucose-lowering medications, have been proven to prevent the onset and progression of DPN (Genuth, 2006), no reliable therapy that has the potential to restore the neurophysiological dysfunction in an advanced stage of DPN has been established (Kobayashi and Zochodne, 2018). In the current study, to suggest a novel pathological mechanism in DPN, we investigated the involvement of ATP-sensitive potassium (K_ATP_) channels, which regulate neuron development and neuronal excitability, in the physiology of the peripheral nervous system (PNS).

Voltage-gated potassium channels are known to suppress neuronal excitatory activity in the central and peripheral nervous systems and to be neuroprotective through avoiding excitotoxicity (Deng et al., 2005, 2011; Malin and Nerbonne, 2002). Another type of potassium channel, the inward-rectifying potassium channel, also controls the stability of resting membrane potentials and the excitability of neurons. Among the inward-rectifying potassium channels, the K_ATP_ channels attract attention for their association with metabolic diseases. The K_ATP_ channels are hetero-octamers formed by four pore-forming inward rectifier channel subunits, KIR6.1 (KCNJ8) or KIR6.2 (KCNJ11), and four sulfonylurea receptor subunits, SUR1 (ABCC8) or SUR2 (ABCC9). Of the various types of K_ATP_ channels, the K_ATP_ channel composed of KIR6.2 and SUR1 is most known for its pivotal role in the regulation of glucose-responsive insulin secretion from pancreatic beta cells. The K_ATP_ channels of beta cells set a plasma membrane potential by opening the channels in the resting state. Under increased levels of plasma glucose, the channels close, depolarize the membrane potential, and induce an insulin release following the opening of voltage-gated calcium channels (Lang et al., 2011). In addition to the pancreas, it has been proven that K_ATP_ channels are expressed in a wide variety of tissues including the central nervous system (CNS) (Mourre et al., 1990) in which glucose is the major energy-yielding substrate. As K_ATP_ channels are involved in the peripheral analgesic pathway (Sachs et al., 2004), several analgesic substances including diclofenac, sodium nitroprusside, and morphine activate K_ATP_ channels to induce their systemic antinociceptive effect (Alves et al., 2004; Cunha et al., 2010; Ortiz et al., 2012). The depolarization of the membrane potential induced by glucose leads directly to the hyperexcitability of neurons (Himeno and Nakamura, 2017). Those neurons, so-called glucose-sensing neurons, have recently been investigated as a modulator of glucose homeostasis (Khodai et al., 2018; Labouebe et al., 2016; Stanley et al., 2019). Furthermore, in recent reports, some types of K_ATP_ channels exist on mitochondrial membranes and have critical physiological roles in maintaining intact mitochondrial functions (Coetzee, 2013; Foster et al., 2012). In this context, K_ATP_ channel openers, diazoxide and nicorandil, have been proven to prevent neurodegeneration (Kong et al., 2013; Virgili et al., 2013; Watanabe et al., 2008) and reduce neuronal death in cerebral ischemia of rats (Farkas et al., 2006). It has been indicated that these K_ATP_ channel openers mimic ischemic preconditioning in cardiomyocytes (Coetzee, 2013) and neurons in the CNS (Kis et al., 2003) and provide beneficial effects to these cells.

Neuronal damage in the CNS has been reported after ischemic stress increased in *Kir6.2* knockout mice (Sun et al., 2006), in which a plasma membrane potential was depolarized (Miki et al., 1998). It has also been proven in the PNS that, after 24 h, hyperglycemia induces depolarization of the resting membrane potential in neurons through closing the K_ATP_ channel and through mechanical hyperalgesia (de Campos Lima et al., 2019). *KCNJ11* activating mutations in humans are associated with developmental delay, epilepsy, neonatal diabetes, and other neurological features (Gloyn et al., 2006). Regarding the other potassium inward rectifier channel KCNJ10, patients suffering from *KCNJ10* mutations develop EAST syndrome, which is characterized by epilepsy, ataxia, sensorineural deafness, and renal tubulopathy (Reichold et al., 2010). In brief, K_ATP_ channels may have the potential to change in activity and maintain the integrity of both CNS and PNS neurons.

Here, to clarify the physiological significance of K_ATP_ channels in the PNS, we evaluated distributions of proteins that compose K_ATP_ channels in mouse dorsal root ganglion (DRG) neurons. Then, we determined the effects of K_ATP_ channel blockers/activator on the neurite outgrowth of cultured DRG neurons. Thereafter, we evaluated the functional and morphological changes of the PNS in *Kir6.2* knockout mice, in which the aberrant K_ATP_ channel causes loss of responsiveness to glucose and sulfonylurea.

## Results

### Expressions of components of K_ATP_ channels in DRG

**Figure 1:**
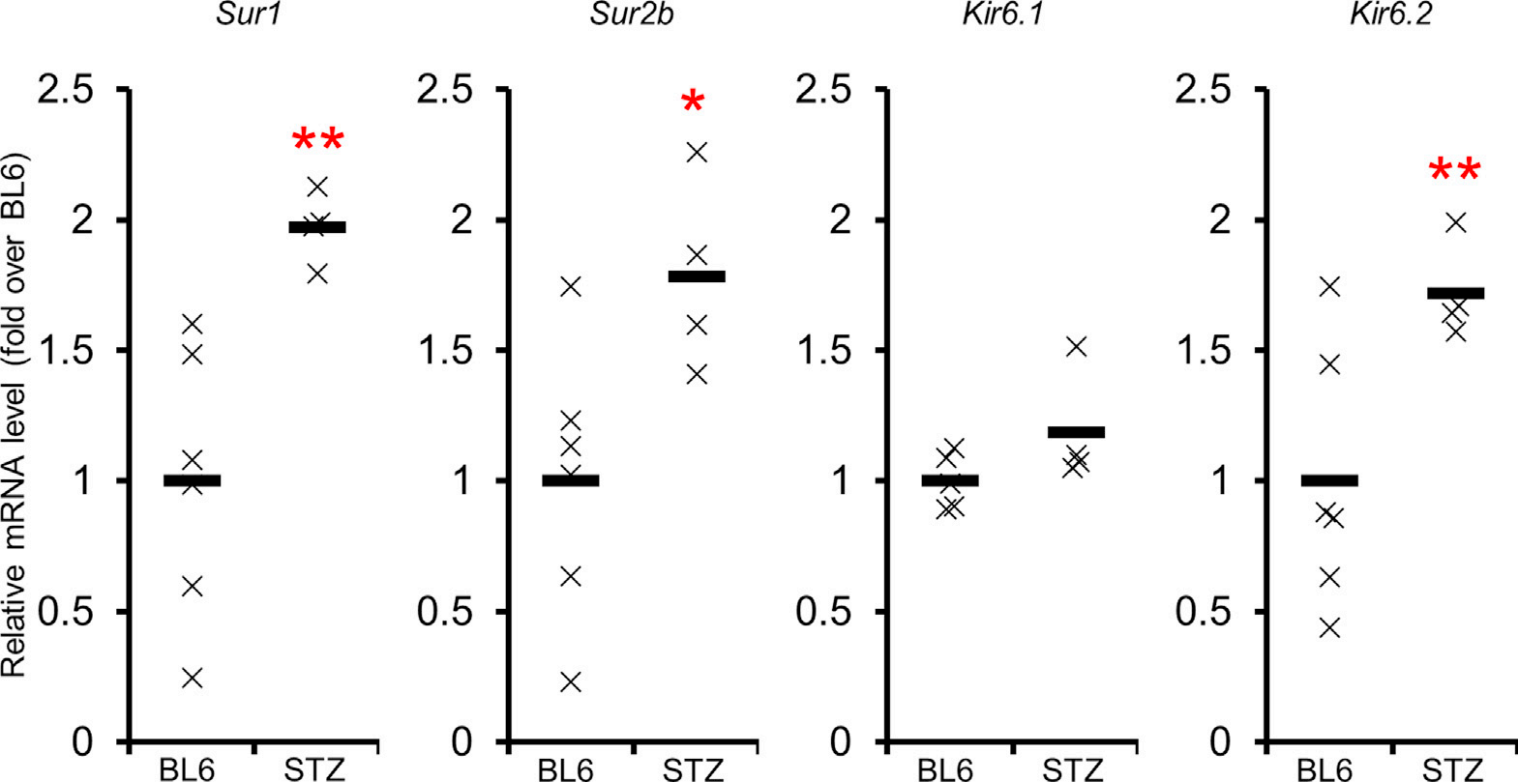
Real-time qPCR of K_ATP_ channels in dorsal root ganglia (DRG) of wild-type or diabetic mice Expression levels of K_ATP_ channel component proteins in DRG of C57BL6/J (BL6) or streptozotocin (STZ)-induced diabetic mice. *Sur1*, *Sur2B*, *Kir6.2*, and *Kir6.1* were expressed in the DRG of both types of mice. The expression levels of *Sur1*, *Sur2B*, and *Kir6.2* in STZ-induced diabetic mice were significantly higher than those in BL6 mice. SUR, sulfonylurea receptor; Kir, inward rectifier potassium channel; BL6, C57BL6/J mouse; STZ, streptozotocin-induced diabetic mouse. ∗: p < 0.01 versus BL6, ∗∗: p < 0.001 versus BL6.

The reverse transcriptase-PCR (qPCR) experiments revealed that mRNA of K_ATP_ channel subunits was expressed in murine DRG (Figure 1). Both pore domains *Kir6.1* and *Kir6.2* were detected in DRG. The expression levels of *Kir6.2* in streptozotocin (STZ)-induced diabetic mice were significantly higher than that of non-diabetic C57BL/6 (BL6) (p < 0.01). The expression level of *Kir6.1* in C57BLKS/J- + Lepr^db^/+Lepr^db^ (db/db) diabetic mice was also significantly higher than that of C57BLKS/J (BLKS) mice in their genetic background (p < 0.001) and that of their C57BLKS/J-m+/+Lepr^db^ (db/+) control littermates (p < 0.005) (Figure S1). Three SURs are known in mice: SUR1, SUR2A, and SUR2B. Among these three SURs, the expression of *Sur2A* was not verified in DRG (data not shown). The expression of *Sur1* and *Sur2b* increased in STZ-induced diabetic mice (p < 0.01 and p < 0.05, respectively) compared with BL6 (Figure 1).

**Figure 2:**
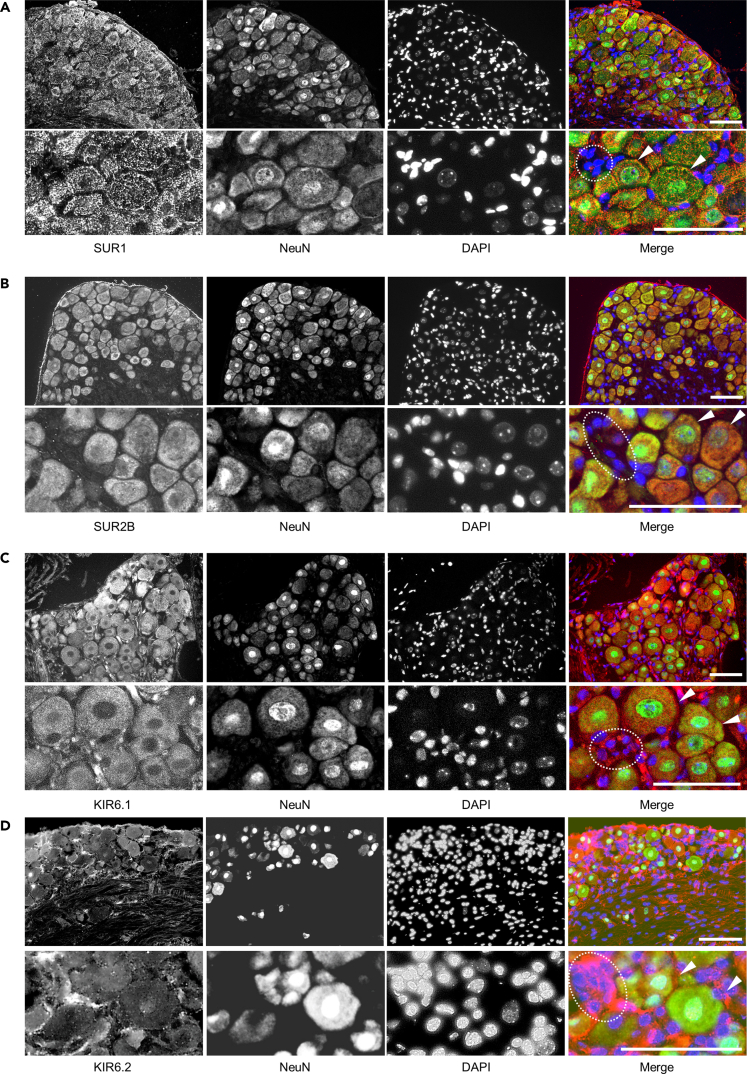
Immunohistochemistry of K_ATP_ channel proteins in dorsal root ganglia (DRG) in C57BL6/J mice SUR1 (A), SUR2B (B), KIR6.1 (C), and KIR6.2 (D) were detected in DRG neurons and non-neuronal cells in DRG. Merge images represent NeuN (green), DAPI (blue), and each component protein of K_ATP_ channels (red). Lower panels represent high-power views in the corresponding upper panels. White dotted circles, non-neuronal cells; white arrowheads, neurons. Scale bar: 100 μm.

Immunostaining revealed the protein expression of SUR1, SUR2B, KIR6.1, and KIR6.2 in neurons and a part of non-neuronal cells (Figures 2A–2D). The protein expression of SUR2A was not able to be detected (data not shown).

### Neurite lengths of DRG neurons were shortened with K_ATP_ channel closers and extended with its opener

**Figure 3:**
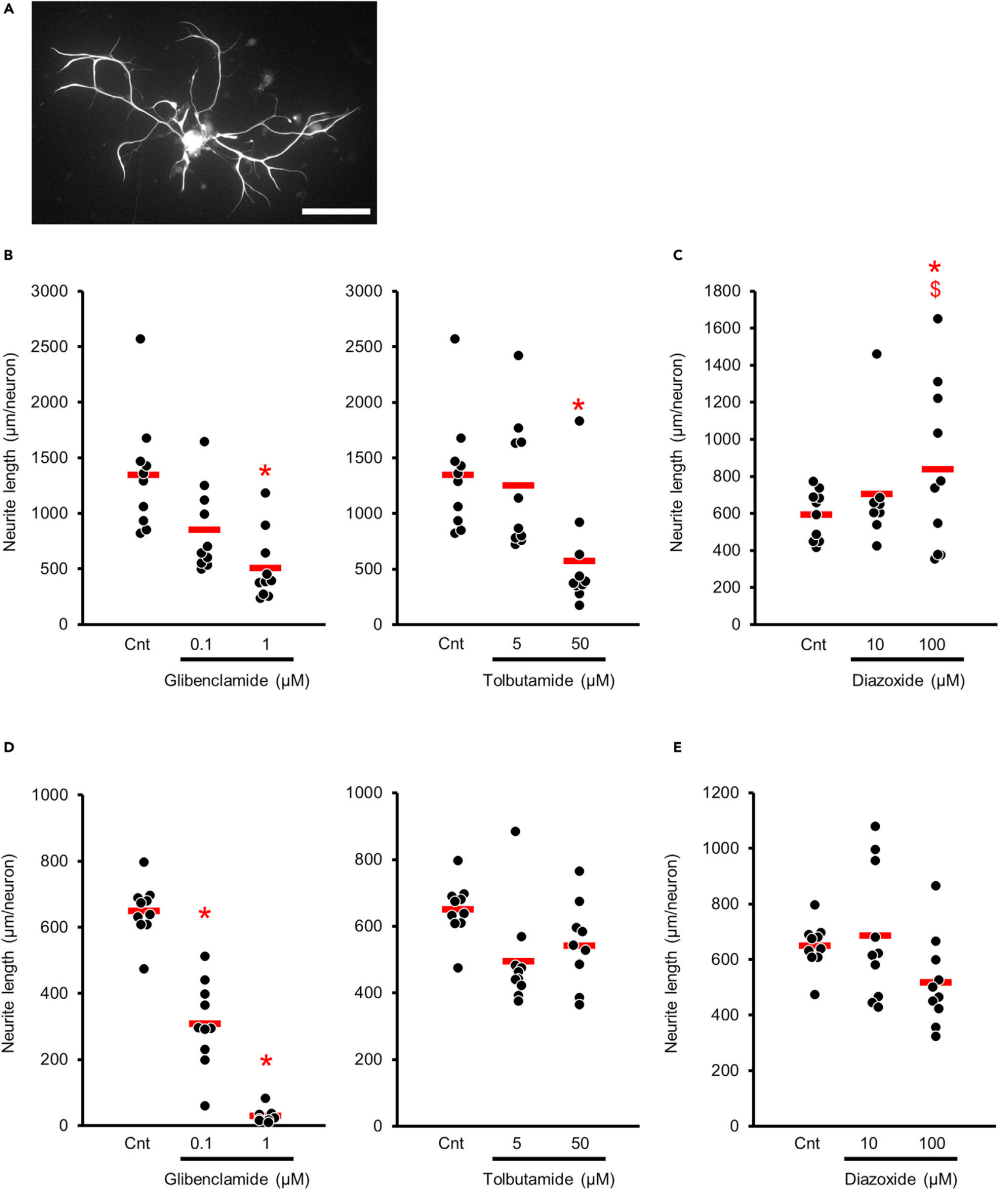
Neurite lengths of DRG neurons were shortened with K_ATP_ channel closers and extended with its opener (A) A representative image of DRG neuron and its neurites obtained from C57BL6/J mouse cultured without any modifiers of K_ATP_ channels. Scale bar: 100 μm. (B) Neurite lengths of DRG neurons were shortened by administering K_ATP_ channel closers, glimepiride and tolbutamide (glibenclamide: control 1,349.1 ± 490.7 μm/neuron, 0.1 μmol/L 857.0 ± 383.1, 1 μmol/L 511.4 ± 311.2; tolbutamide: control 1,349.1 ± 490.7, 5 μmol/L 1,315.1 ± 551.52, 50 μmol/L 576.9 ± 462.6; *n* = 10 in each group). After culturing for 24 h, neurite lengths of each neuron visualized by immunostaining with anti-neurofilament antibodies were quantified. The horizontal red line in each graph represents the mean value. (C): Neurite outgrowth of neurons was accelerated by administrations of the K_ATP_ channel opener diazoxide (control 595.5 ± 125.9, 10 μmol/L 705.0 ± 296.1, 100 μmol/L 840.0 ± 427.9; *n* = 8 or 10 in each group). Cnt, control. ∗: p < 0.05 versus control, $: p < 0.05 versus 10 μmol/L diazoxide. (D) Neurite outgrowths in DRG neurons obtained from *Kir6.2*^−/−^ mice. Glibenclamide but not tolbutamide decreased neurite outgrowths (control 651.0 ± 78.5, 0.1 μmol/L glibenclamide 309.7 ± 122.9, 1 μmol/L glibenclamide 31.0 ± 21.5, 5 μmol/L tolbutamide 495.5 ± 139.7, 50 μmol/L tolbutamide 542.4 ± 115.7; *n* = 10 in each group). ∗: p < 0.05 versus control. (E) Neurite outgrowths in DRG neurons obtained from *Kir6.2*^−/−^ mice. Diazoxide had no effects on neurite outgrowths (control 651.0 ± 78.5, 10 μmol/L diazoxide 688.0 ± 227.3, 100 μmol/L diazoxide 518.9 ± 151.6; *n* = 10 in each group). ∗: p < 0.05 versus control. Data are represented as mean ± SD.

Since axonal degradation precedes depletion of DRG neurons in DPN, neurite outgrowths in a primary culture of DRG neurons are utilized to assess the favorable or unfavorable effects in DPN under various circumstances including the supplementation of chemical compounds or cell stress. Therefore, the neurite outgrowths in DRG cultures were examined to reveal the influences of K_ATP_ channel activity on axonal regeneration (Figure 3A). K_ATP_ channel inhibition with its closers, glimepiride (non-selective blocker of SUR1 and SUR2) and tolbutamide (selective blocker of SUR1), shortened neurite lengths of DRG neurons (Figure 3B). In contrast, K_ATP_ channel activation with its opener diazoxide elongated neurite lengths (Figure 3C).

On the other hand, tolbutamide and diazoxide had no effects on the neurite outgrowths in DRG neurons obtained from *Kir6.2*-deficient (*Kir6.2*^−/−^) mice (Figures 3D and 3E). However, glibenclamide decreased neurite outgrowths even in DRG neurons from *Kir6.2*^−/−^ mice (Figure 3D).

### *Kir6.2*-deficient mice showed neurological dysfunction in the PNS

**Figure 4:**
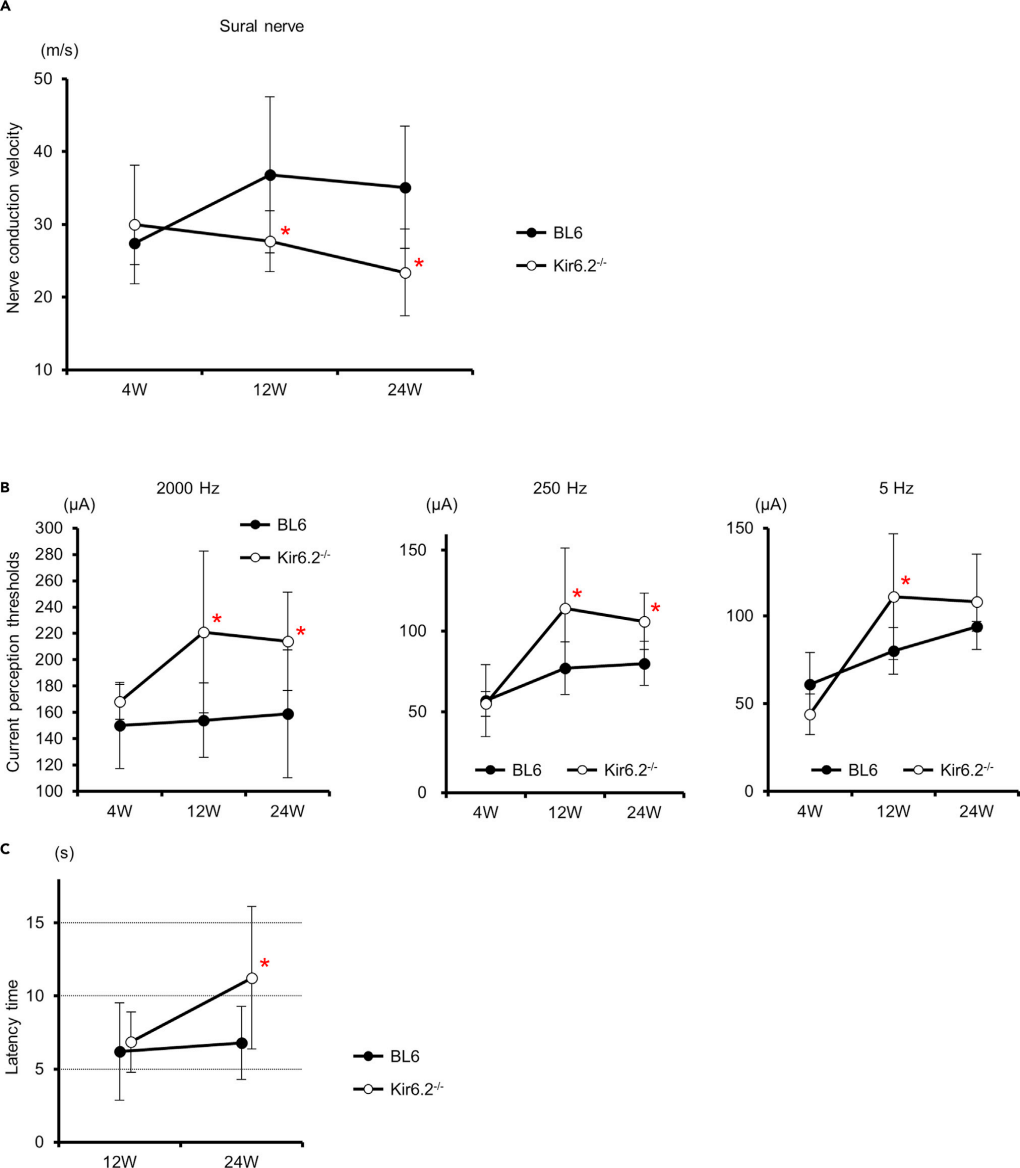
Neurological dysfunction in the peripheral nervous system of *Kir6.2*^−/−^ mice (A) Sensory nerve conduction velocities of *Kir6.2*^−/−^ mice declined with age (12 weeks: BL6 36.8 ± 10.7 m/s, *Kir6.2*^−/−^ 27.7 ± 4.2; 24 weeks: BL6 35.1 ± 8.2, *Kir6.2*^−/−^ 23.4 ± 6.0; *n* = 4–7 in each group). Solid circles, BL6 mice; open circles, *Kir6.2*^−/−^ mice; BL6, C57BL6/J mouse; W, week-old. ∗: p < 0.05 versus BL6 mice. (B) Current perception thresholds (CPTs) of plantar were determined with Neurometer. Electric currents with frequencies of 2,000, 250, and 5 Hz stimulated predominantly Aβ, Aδ, and C fiber, respectively. All CPTs in *Kir6.2*^−/−^ mice increased with age compared with those in BL6 mice at 12 weeks old (2,000 Hz: BL6 153.5 ± 28.3 μA, *n* = 15, *Kir6.2*^−/−^ 221.1 ± 61.4, *n* = 6; 250 Hz: BL6 77.2 ± 16.2 μA, *n* = 10, *Kir6.2*^−/−^ 113.8 ± 37.3, *n* = 8; 5 Hz: BL6 79.5 ± 13.2 μA, *n* = 10, *Kir6.2*^−/−^ 111.4 ± 35.9, *n* = 7). CPTs for 2,000- or 250-Hz stimuli were still impaired in *Kir6.2*^−/−^ at 24 weeks of age (2,000 Hz: BL6 158.7 ± 48.4 μA, *n* = 15, *Kir6.2*^−/−^ 213.9 ± 37.5, *n* = 6; 250 Hz: BL6 80.1 ± 13.6 μA, *n* = 15, *Kir6.2*^−/−^ 103.6 ± 17.5, *n* = 6; 5 Hz: BL6 94.5 ± 26.7 μA, *n* = 15, *Kir6.2*^−/−^ 107.8 ± 27.1, *n* = 6). W, week-old; solid circles, BL6 mice; open circles, *Kir6.2*^−/−^ mice. ∗: p < 0.05 versus BL6 mice. (C) Thermal plantar test. There was no significant difference in thermal perception thresholds between *Kir6.2*^−/−^ mice and BL6 mice at age 12 weeks (BL6 6.2 ± 3.3 s, *n* = 15, *Kir6.2*^−/−^ 6.5 ± 2.1, *n* = 7). However, the thresholds increased in *Kir6.2*^−/−^ mice compared with BL6 mice at 24 weeks of age (BL6 6.8 ± 2.5 s, *n* = 19, *Kir6.2*^−/−^ 11.0 ± 5.0, *n* = 11). W, week-old; solid circles, BL6 mice; open circles, *Kir6.2*^−/−^ mice. ∗: p < 0.05 versus BL6 mice. Data are represented as mean ± SD.

We evaluated the physiological functions of the PNS in *Kir6.2*^−/−^ mice. Sensory nerve conduction velocities (SNCVs) in sural nerves decreased in *Kir6.2*^−/−^ mice compared with BL6 mice at age 12 and 24 weeks (12 weeks: BL6 36.8 ± 10.7 m/s, *Kir6.2*^−/−^ 27.7 ± 7.1; 24 weeks: BL6 35.1 ± 8.2, *Kir6.2*^−/−^ 23.4 ± 6.0; *n* = 4–7 in each group) (Figure 4A). Current perception thresholds (CPTs) using electrical stimuli were examined to evaluate perceptions of planta pedis. All three types of CPTs, examined using stimuli with three different frequencies, were exacerbated in *Kir6.2*^−/−^ compared with BL6 mice at 12 weeks of age (2000 Hz: BL6 153.5 ± 28.3 μA, *Kir6.2*^−/−^ 221.1 ± 61.4; 250 Hz: BL6 77.2 ± 16.2 μA, *Kir6.2*^−/−^ 113.8 ± 37.3; 5 Hz: BL6 79.5 ± 13.2 μA, *Kir6.2*^−/−^ 111.4 ± 35.9; *n* = 6–15 in each group; p < 0.05 compared with BL6 mice in each group) (Figure 4B). CPTs for 2,000- or 250-Hz stimuli were still impaired in *Kir6.2*^−/−^ at 24 weeks of age (2,000 Hz: BL6 158.7 ± 48.4 μA, *Kir6.2*^−/−^ 213.9 ± 37.5; 250 Hz: BL6 80.1 ± 13.6 μA, *Kir6.2*^−/−^ 103.6 ± 17.5; 5 Hz: BL6 94.5 ± 26.7 μA, *Kir6.2*^−/−^ 107.8 ± 27.1; *n* = 6–10 in each group; p < 0.05 compared with BL6 mice in stimuli with 2,000 or 250 Hz). In addition, thermal perceptions decreased in 24-week-old *Kir6.2*^−/−^ mice in the thermal plantar test, which was performed to provide supportive evidence with real physical stimuli (BL6 6.8 ± 2.5 s, *Kir6.2*^−/−^ 11.0 ± 5.0; *n* = 11 or 19 in each group; p < 0.05) (Figure 4C).

### Unmyelinated fibers in the sural nerves were reduced in *Kir6.2*-deficient mice

**Figure 5:**
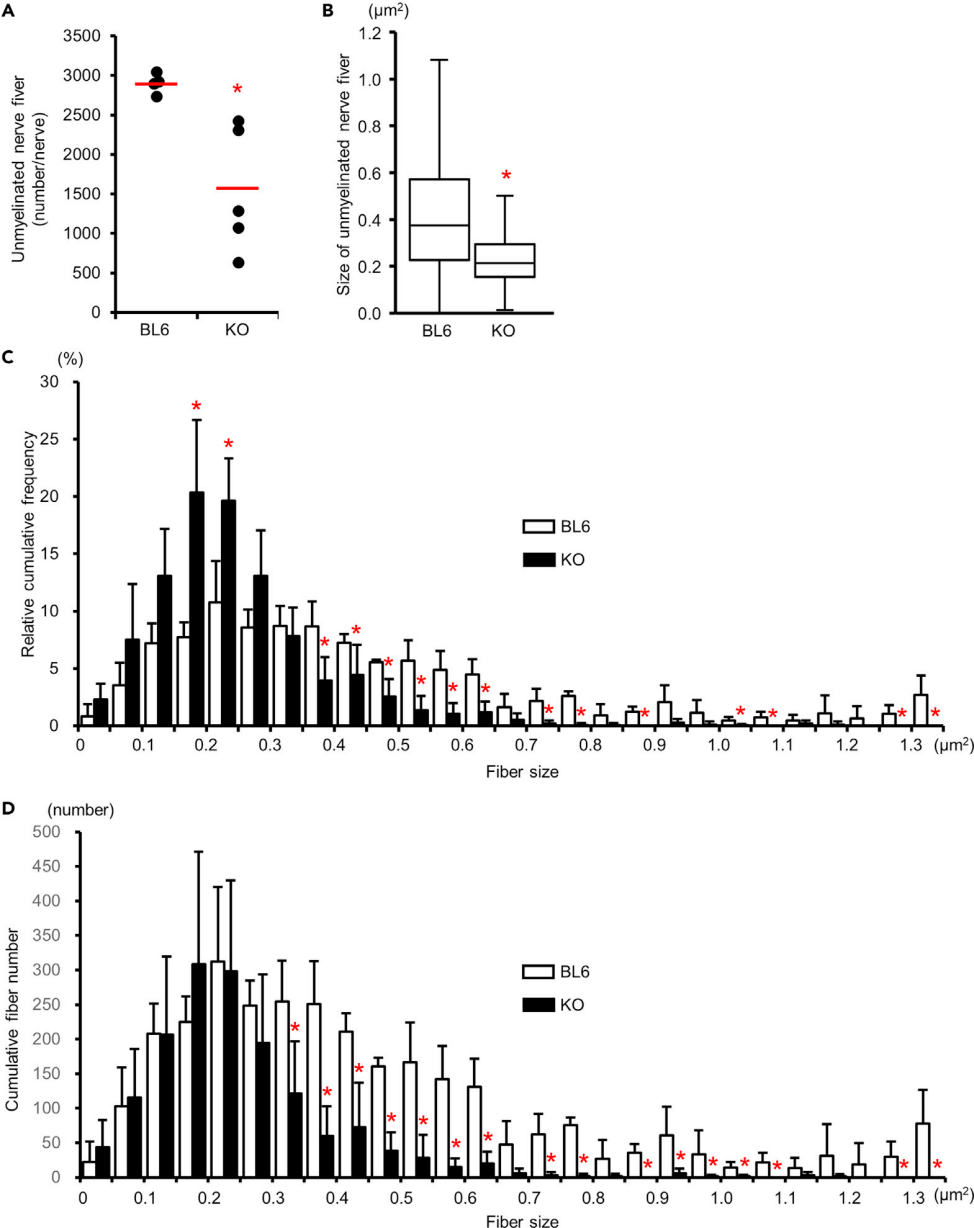
Morphology of unmyelinated nerve fibers in the sural nerve of *Kir6.2*^−/−^ mice A: Total numbers of unmyelinated fibers in the whole sural nerve decreased in *Kir6.2*^−/−^ mice compared with C57BL6J mice. N = 5 in *Kir6.2*^−/−^ mice, n = 4 in C57BL/6J mice. BL6, C57BL/6J mice; KO, *Kir6.2*^−/−^ mice. ∗: *p* < 0.05 compared with C57BL/6J mice. Red horizontal lines: mean values. Black closed circles: a value in each mouse. (B) Mean size of unmyelinated fibers in *Kir6.2*^−/−^ mice decreased compared with C57BL/6J mice. N = 1,154 in *Kir6.2*^−/−^ mice, n = 670 in C57BL/6J mice. ∗: p < 0.05 compared with C57BL/6J mice. In the boxplots, the horizontal line in the box represents the median. The edges of the boxes are the 25^th^ and 75^th^ quartile values. The ends of the whiskers are minimum and maximum values. (C) A frequency histogram of unmyelinated fibers in the sural nerve. Open bars represent C57BL/6J mice. Solid bars are *Kir6.2*^−/−^ mice. N = 5 in *Kir6.2*^−/−^ mice, n = 4 in C57BL/6J mice. ∗: p < 0.05 compared with C57BL/6J mice. (D) A histogram of the estimated number in each range of fiber size in the whole sural nerve. Open bars represent C57BL/6J mice. Solid bars are *Kir6.2*^−/−^ mice. N = 5 in *Kir6.2*^−/−^ mice, n = 4 in C57BL/6J mice. ∗: p < 0.05 compared with C57BL/6J mice. Data are represented as mean±SD.

The sural nerve morphometry obtained from 6-month-old mice showed no significant difference between *Kir6.2*^−/−^ and BL6 mice. Therefore, we have extended the period of investigation to 18 months old. In the sural nerve morphometry using electron microscopy, the number of unmyelinated nerve fibers decreased in *Kir6.2*^−/−^ mice compared with BL6 mice (Table 1). On the other hand, the number of myelinated nerve fibers showed no reduction in *Kir6.2*^−/−^ mice. The number and size of axons decreased in unmyelinated fibers (Figures 5A and 5B) but not in myelinated fibers (Table 1). The number and occupancy of Remak bundles, ensheathing multiple unmyelinated axons, in the sural nerve also decreased in *Kir6.2*^−/−^ mice (Table 1). In addition, the number and occupancy of C-fibers in Remak bundles decreased in *Kir6.2*^−/−^ mice (Table 1). Morphometry of myelinated fibers in the sural and sciatic nerves (Table S1) showed that *g*-ratio and the total number of Schwann cell nuclei indicated no significant difference between these two types of mice. These results indicate that Schwann cell dysfunction may not be responsible for the morphometric changes in *Kir6.2*^−/−^ mice.

**Table 1:** Quantification of morphometry in the sural nerve using electron microscopy

	C57BL6/J (n = 4)		*Kir6.2*^−/−^ (n = 5)		P
	Mean	SD	Mean	SD	
Whole nerve size (μm^2^)	22,429.3	4,698.6	17,158.5	2,223.5	*0.091*
Total fibers (no./nerve)	3,526.0	233.9	1,927.3	724.2	0.007^∗∗^
Myelinated fibers (no./nerve)	623.2	190.2	379.3	115.1	0.074
% Myelinated fibers	17.4	4.2	22.4	9.2	0.403
Myelinated fiber density (10^3^/mm^2^)	27,419.3	2,957.0	21,795.8	4,935.4	0.120
Myelinated fiber size (μm^2^)	21.7	1.5	21.1	4.9	0.820
Mean myelin area (μm^2^)	11.9	1.1	11.7	2.7	0.864
Mean myelin thickness (μm)	0.86	0.06	0.84	0.09	0.807
Mean axon area (μm^2^)	9.8	0.6	9.4	2.2	0.779
*g*-ratio	0.64	0.01	0.64	0.02	0.893
Unmyelinated fibers (no./nerve)	2902.8	109.0	1,547.9	703.8	0.012^∗^
% Unmyelinated fibers	82.6	4.2	77.6	9.2	0.403
Unmyelinated fiber density (10^3^/mm^2^)	110.3	36.0	93.4	49.1	0.317
Unmyelinated fiber size (μm^2^)	0.47	0.08	0.24	0.04	0.001^∗∗^
Remak bundles (no./nerve)	250.9	14.0	163.5	34.7	0.004^∗∗^
Bundle occupancy in nerve (%)	9.0	0.6	5.4	2.5	0.044^∗^
Mean Remak bundle size (μm^2^)	5.9	2.4	4.2	1.3	0.012^∗^
C-fibers per Remak bundle (no.)	9.6	3.3	6.6	3.9	0.001^∗∗^
C-fiber occupancy in a bundle (%)	69.7	4.0	52.7	4.9	0.002^∗∗^

∗: p < 0.05, ∗∗: p < 0.01.

### Unmyelinated nerve fibers degenerated in the sural nerve of *Kir6.2*-deficient mice

**Figure 6:**
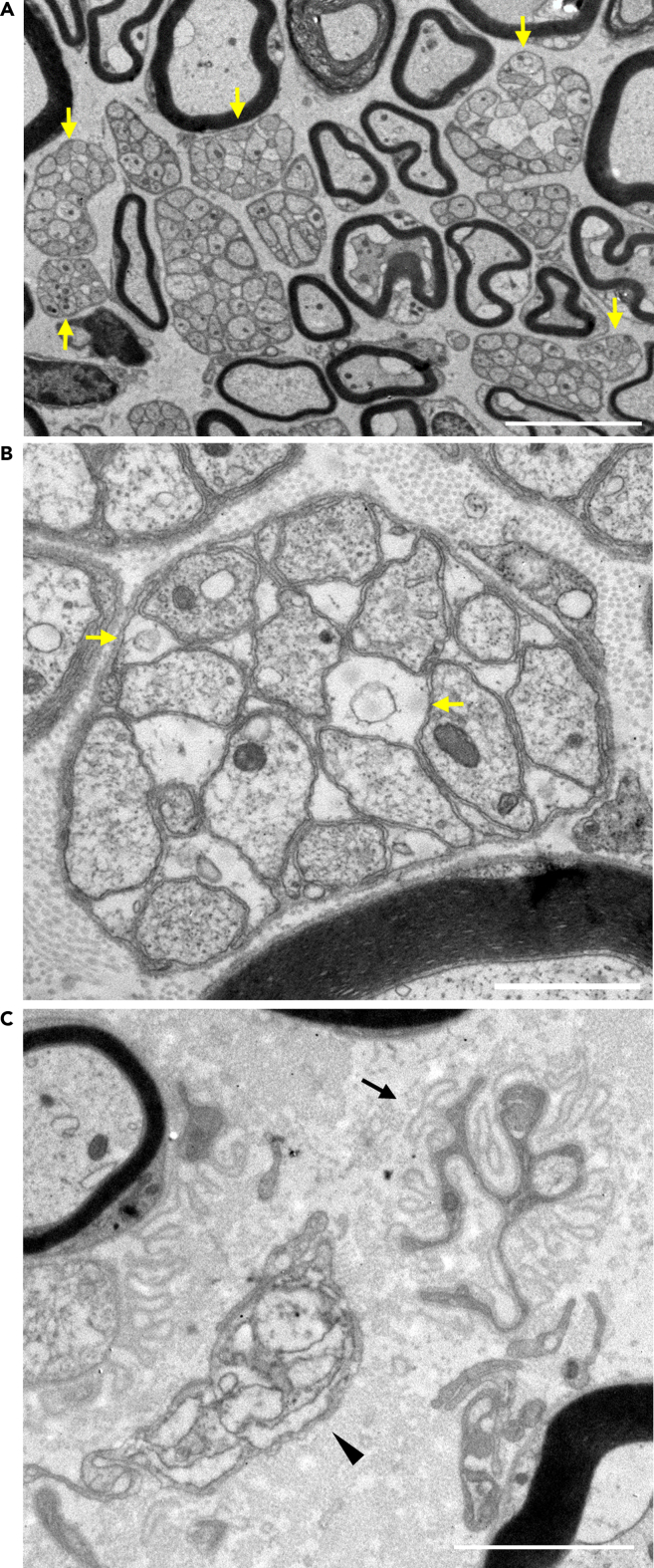
Ultrastructural changes of unmyelinated nerve fibers in the sural nerve of *Kir6.2*^−/−^ mice (A) Remak bundles include an empty area among axons (arrows). Scale bar: 5 μm. (B) In part of the empty area, there is an astral scar implying a degenerated axon (arrows). Scale bar: 1 μm. (C) Remak bundle with a few axons (arrowhead) and a denervated Schwann cell profile (arrow). Scale bar: 2 μm.

The histogram analysis using percentages of unmyelinated fiber size distribution showed a shift toward smaller sizes in *Kir6.2*^−/−^ mice (Figure 5C). As the number of unmyelinated fibers decreased, we also analyzed absolute fiber numbers in the entire sural nerve for histogram analysis. The histogram using absolute unmyelinated fiber numbers in each size range revealed that this shift was caused by the reduction of numbers in medium and large-sized unmyelinated fibers (Figure 5D). A characteristic ultrastructural change was that many Remak bundles include empty gaps among axons (Figure 6A). In some of these empty gaps, possibly caused by inflated Schwann cell tongues, there is an astral scar, implying a degenerated axon (Figure 6B). Bundles were involved heterogeneously in the unmyelinated axonal loss; an affected bundle was either located next to (Figures 6A and S2), or connected with, an intact bundle (Figure S3). Furthermore, *Kir6.2*^−/−^ mice exhibited frequent denervated Schwann cell profiles (Figures 6C and S2).

### Myelinated nerve fibers exhibited degeneration and regeneration in the peripheral nerves of *Kir6.2*-deficient mice

The size-frequency histogram analysis of myelinated fibers in the sural nerve using percentages of axon size distribution showed no significant differences between *Kir6.2*^−/−^ and C57BL6/J mice (Figure S4A). However, the histogram analysis for absolute axon numbers in the entire sural nerve revealed a significant decrease in small to medium-sized axons (Figure S4B). The histogram using myelin size showed no significant difference between these two mice (Figure S4C).

**Figure 7:**
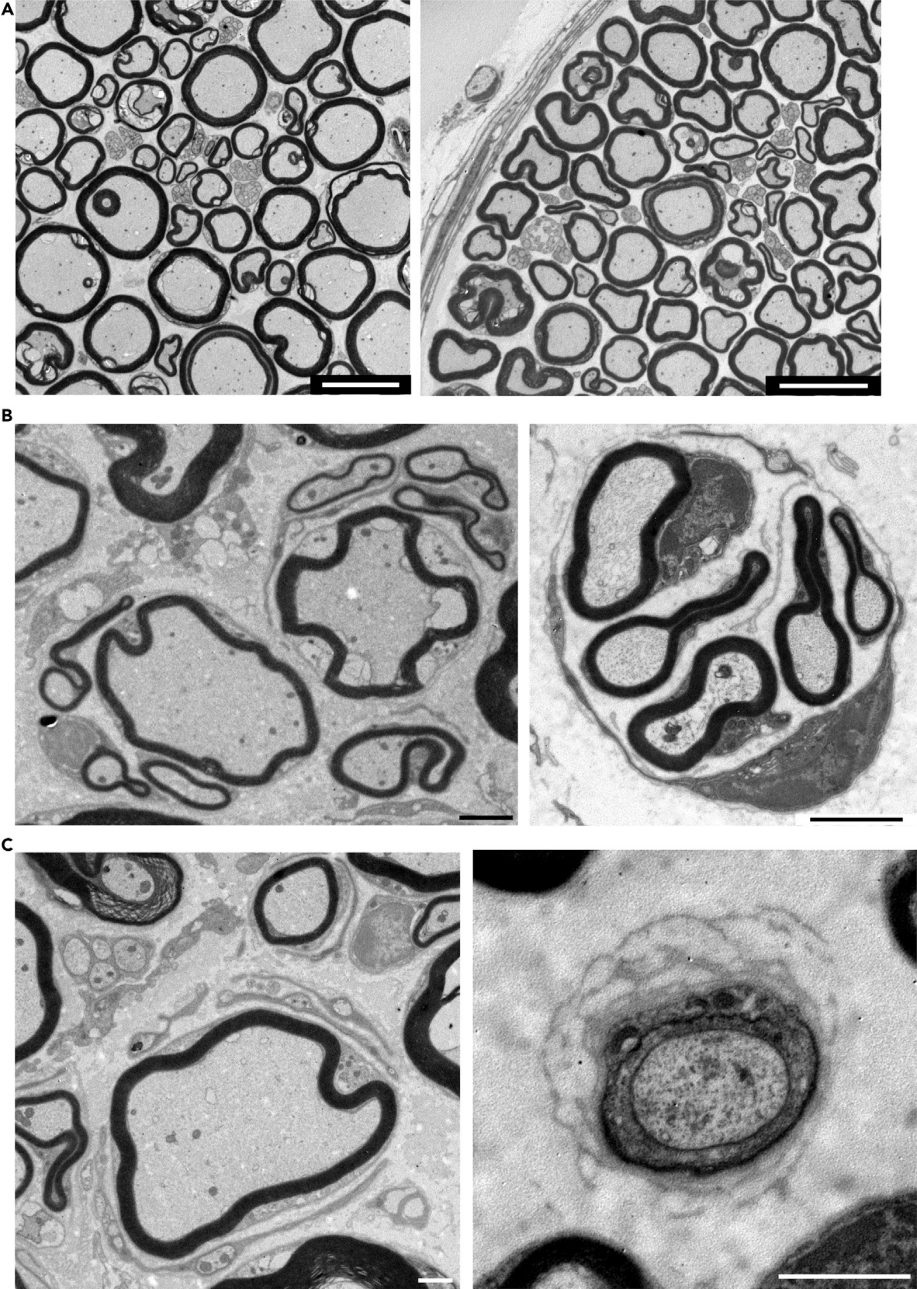
Occasional signs of degeneration in myelinated fibers of *Kir6.2*^−/−^ mice (A) Electron micrograph of transverse sections through the sciatic (left) and sural (right) nerves of *Kir6.2*^−/−^ mouse. Scale bars: 10 μm. (B) Clusters of regenerating myelinated fibers in the sciatic nerve of *Kir6.2*^−/−^ mice. Scale bars: 2 μm. (C): Onion bulb-like structures in the sciatic (left) and sural (right) nerve of *Kir6.2*^−/−^ mice. Scale bars: 1 μm.

Electron microscopy showed that most myelinated fibers had a normal appearance in the sciatic and sural nerves of *Kir6.2*^−/−^ mice (Figure 7A). However, the sciatic nerve frequently contained clusters of regenerating myelinated fibers (Figures 7B and S5). Occasionally, onion bulb-like lamellated structures were observed (Figures 7C and S5), which are concentric layers of Schwann cell processes caused by repetitive segmental demyelination and regeneration of myelin.

## Discussion

The major findings of the current study are (1) DRG neurons express components of K_ATP_ channels at both transcript and protein levels and the levels of certain transcripts increased in mice with hyperglycemia; (2) K_ATP_ channel inhibition with its closers shortens, but K_ATP_ channel activation with its opener elongates, neurite outgrowths in the primary culture of DRG neurons; (3) *Kir6.2*^−/−^ mice present the dysfunction of electrophysiology and sensory perception in the peripheral nerves of the lower extremities; and (4) *Kir6.2*^−/−^ mice present ultrastructural abnormalities in peripheral somatosensory nerves.

It has been proven that some components of K_ATP_ channels were expressed (Zoga et al., 2010) and activation of K_ATP_ channels attenuated hyperexcitability in DRG neurons of rats (Du et al., 2011; Luu et al., 2019). In this context, we revealed the transcript levels of K_ATP_ components in murine DRG. We also showed that the constitutive proteins of K_ATP_ channels existed in the DRG using immunostaining. Furthermore, these expression levels in STZ-induced hyperglycemic or db/db mice were increased compared with those in non-diabetic mice. It has been reported in a previous paper that the increased K_ATP_ channel density regulated the protective property against hypoxia in cardiomyocytes (Ranki et al., 2002). Therefore, although the significance of altered expression levels in K_ATP_ channel components in diabetic mice has not yet been clarified, the increase might strengthen the neuroprotective properties of the K_ATP_ channel in DPN. However, as the functional change of K_ATP_ channels cannot be assessed precisely by their expression levels, we will plan to investigate the electrical properties of DRG neurons in diabetic mice in the future.

The effects of K_ATP_ channel activation or inhibition on axonal outgrowth were investigated in primary cultures of mouse DRG neurons. As dying-back axonal degeneration is a prominent pathology in DPN, it is currently estimated that the distal ends of nerve fibers decline in the early stage of DPN. Therefore, the *ex vivo* experiment evaluating neurite outgrowth of DRG neurons was utilized to reproduce a failure of distal end formation in DPN. In the current study, the neurite outgrowth was shortened by the channel closers sulfonylureas, which are widely prescribed to patients with diabetes as hypoglycemic agents. Conversely, the channel opener diazoxide significantly elongated the neurite lengths. These results suggest that the inhibition of the K_ATP_ channel may exacerbate neural degeneration, but the activation prevents degeneration. It has been reported that membrane excitability in the axonal growth cones of embryonic neurons influences axonal growth (Huang et al., 2017). According to the report, increased membrane excitability inhibited axonal growth. Therefore, in the current study, the change in neuronal excitability might explain the alterations in neurite outgrowth.

Although glibenclamide irreversibly binds to SUR1 and SUR2A and has strong effects to accelerate insulin secretion, tolbutamide reversibly binds to SUR1 and inhibits SUR1 but not SUR2A (Gribble and Reimann, 2003). We should consider the possibility that the selective affinity of tolbutamide may induce different behaviors on DRG neurons. Given tolbutamide produced no alteration of neurite outgrowths in DRG neurons obtained from *Kir6.2*^−/−^ mice, the selectivity of affinity to SURs might be an important factor in whether each K_ATP_ channel closer has neuroprotective or neurotoxic effects on DRG neurons.

K_ATP_ channel openers, diazoxide and nicorandil, have been proven to prevent neurodegeneration (Kong et al., 2013; Virgili et al., 2013; Watanabe et al., 2008) and reduce neuronal death in cerebral ischemia of rats (Farkas et al., 2006). It has also been published that nicorandil activated endothelial nitric oxide synthase and provided neuroprotective effects as a consequence of reducing oxidative stress on hypoperfusion-induced dementia model mice (Gupta et al., 2016). Although another K_ATP_ channel opener, diazoxide, acts on both SUR1 and SUR2 and inhibits insulin secretion, nicorandil activates only SUR2 and has no influence on insulin secretion. Therefore, nicorandil is widely used as an anti-anginal drug (Moreau et al., 2000) because nicorandil has been proven to have a myocardial protective effect at the onset of ischemia and reduce cardiovascular deaths (Horinaka et al., 2010) via its preconditioning effect (IONA Study Group, 2002; Ishii et al., 2007). Currently, however, no research has suggested long-term usefulness of nicorandil in human diseases of the PNS. We would like to investigate the neuroprotective effects of nicorandil against DPN in clinical settings in the future.

Finally, we used *Kir6.2*^−/−^ mice to assess the chronic effects of the K_ATP_ channel on the PNS. In Parkinson's disease, which is one of the neurodegenerative diseases of the central nervous system, activation or closure of K_ATP_ channels is known to promote the death of dopamine neurons (Liss et al., 2005). It has also been reported that the expression of SUR1 is increased in dopamine neurons of the substantia nigra in the human brain with Parkinson's disease (Schiemann et al., 2012). Given these facts, it may be suggested that the increased expression of K_ATP_ channel components may conversely imply a decrease in channel function. In addition, we previously reported that the Kir6.2−/− mice showed depolarized membrane potential and were susceptible to hypoxia-induced seizure (Yamada et al., 2001), suggesting that excitotoxicity may be involved in neurons of these mice. Given the excitotoxicity induced by K_ATP_ channel dysfunction and considering the physiological response of K_ATP_ channels to glucose and sulfonylureas, we evaluated the knockout mice lacking responsiveness to glucose and sulfonylurea as a model that might mimic sustained hyperglycemia or chronic administration of sulfonylureas. Nerve conduction velocities (NCVs) in juvenile *Kir6.2*^−/−^ equaled that of wild-type BL6 mice. However, SNCV significantly decreased at 12–24 weeks of age in *Kir6.2*^−/−^ compared with BL6 mice. In addition, the knockout mice that were 12 weeks of age or older showed dysfunction of sensory perception. These facts indicate that the K_ATP_ channels assembled with KIR6.2 are uncritical for the development of the PNS but imperative to maintain the integrity of the PNS. Electron microscopy revealed a change of ultrastructure in the sural and sciatic nerves. The neural degeneration primarily occurred in axons, not in Schwann cells. In the axonal degeneration, small fibers, especially unmyelinated fibers, decreased. However, there was evidence of axonal de/re-generation of myelinated fibers and de/re-myelination. As the immunostaining images of DRGs showed the expression of K_ATP_ channel in non-neuronal cells and the findings of demyelination were observed by electron microscopy, we should consider that the degeneration observed in the nerves of *Kir6.2*^−/−^ mice may be partly due to the degeneration of Schwann cells. Although these heterogeneous structural changes in the PNS of *Kir6.2*^−/−^ mice are consistent with the mixed pathological features of DPN, which consists of prominent axonopathy and less prominent demyelination (Dyck et al., 1986), we should consider the influence of impaired insulin secretion to neurodevelopment of the knockout mice. Although the levels of blood insulin and glucose were not significantly different in the knockout mice from wild-type mice, glucose-stimulated insulin secretion was impaired in the knockout mice (Miki et al., 1998). As insulin is an important growth factor for neuronal cells, the impairment of insulin secretion might cause the neuronal impairment in the knockout mice.

Here we verified the significant role of K_ATP_ channels in the homeostasis of the PNS and suggested that the channel could be a new etiology of peripheral neuropathies through demonstrating the neuroprotective effect of the K_ATP_ channel opener *ex vivo*. In our further research, we will study the detailed modulatory effects of K_ATP_ channels in the PNS under the diabetic state.

### Limitations of the study

First, we should consider the fact that *Kir6.2*^−/−^ mice have a complete deficiency of electrophysiological activity of the K_ATP_ channel in pancreatic beta cells and present neonatal transient hypoglycemia followed by normoglycemia with a partial reduction of glucose-stimulated insulin secretion (Miki et al., 1998). Therefore, the degeneration of peripheral nerves might be caused by transient hypoglycemia and subsequent impairment of insulin secretion. We will address this question using mice with a PNS-specific *Kir6.2* deficiency in the future.

Second, we have not explained the discrepancy between the cellular hyperexcitability and the dysfunction of sensory perception in 12 weeks of age or older *Kir6.2*^−/−^ mice. Although the absence of K_ATP_ channel should decrease the excitability thresholds of sensory neurons, neurological functions of *Kir6.2*^−/−^ mice showed a decrease in sensitivity compared with wild-type mice. Regarding β-cell function, although these knockout mice transiently exhibited neonatal hypoglycemia, levels of blood insulin and glucose were not significantly different in adult knockout mice from wild-type mice (Miki et al., 1998). Given the fact, it is possible that the knockout mice did not exhibit hyperalgesia in this study because the experiments were conducted after infancy and that they might exhibit transient hyperalgesia during the neonatal period.

Third, although we used *Kir6.2*^−/−^ mice to elucidate the pathophysiology of DPN, there is insufficient evidence of the involvement of DPN and K_ATP_ channels. The molecular mechanisms of insulin secretion involving K_ATP_ channels and the increase of K_ATP_ channel components in DRG of diabetic mice might indicate the relevance of K_ATP_ and DPN. However, no direct evidence has been accumulated. Therefore, we should consider that the *Kir6.2*^−/−^ mice may be applicable to explore other peripheral neuropathies.

## STAR★Methods

### Key resources table

**Table:** 

REAGENT or RESOURCE	SOURCE	IDENTIFIER
**Antibodies**		

Rabbit polyclonal anti-KIR6.2	UC Davis/NIH NeuroMab Facility	Cat# 73-393; RRID: AB_2336908
Rabbit polyclonal anti-KIR6.1	Santa Cruz Biotechnology	Cat# sc-11224; RRID: AB_2296514
Rabbit polyclonal anti-SUR1	Santa Cruz Biotechnology	Cat# sc-5789; RRID: AB_2219760
Rabbit polyclonal anti-SUR2A	Santa Cruz Biotechnology	Cat# sc-32462; RRID: AB_2219765
Rabbit polyclonal anti-SUR2B	Santa Cruz Biotechnology	Cat# sc-5793, RRID: AB_2219773
Donkey anti-Goat IgG (H+L) cross-adsorbed, Alexa Fluor 488	Thermo Fisher Scientific	Cat# A-11055; RRID: AB_2534102
Donkey anti-Goat IgG (H+L) cross-adsorbed, Alexa Fluor 594	Thermo Fisher Scientific	Cat# A-11058; RRID: AB_2534105
Mouse monoclonal anti-Neurofilament H, clone NE14, Alexa Fluor 488 conjugated	Merck Millipore	Cat# MAB5256X; RRID: AB_11212967

**Biological samples**		
Mouse dorsal root ganglia	This paper	N/A
Mouse sciatic nerves	This paper	N/A
Mouse sural nerves	This paper	N/A

**Chemicals, peptides, and recombinant proteins**		

Glibenclamide	Sigma-Aldrich	G0325010
Tolbutamine	Sigma-Aldrich	T0891
Diazoxide	Sigma-Aldrich	D9035

**Experimental models: Organisms/strains**		

Mouse: C57BLKS/J-m+/+Lepr^db^	The Jackson Laboratory	Cat# JAX:000642; RRID:IMSR_JAX:000642
Mouse: C57BLKS/J-+Lepr^db^/+Lepr^db^	The Jackson Laboratory	Cat# JAX:000697; RRID:IMSR_JAX:000697
Mouse: *Kir6.2*-deficient	RIKEN BioResource Center	Cat# RBRC09393, RRID:IMSR_RBRC09393

**Oligonucleotides**		

Primers for K_ATP_ channel subunits, see Table S2	This paper	N/A

**Software and algorithms**		

Image J 1.52a software	National Institutes of Health	N/A
AxonSeg software	MatLab	N/A

### Resource availability

#### Lead contact

Further information and requests for resources and reagents should be directed to and will be fulfilled by the lead contact, Tatsuhito Himeno (thimeno@aichi-med-u.ac.jp).

#### Materials availability

This study did not generate new unique materials.

### Experimental model and subject details

#### Animals

Six models of male house mice were utilized: C57BL/6 (BL6; IMSR Cat# JAX:000664, RRID:IMSR_JAX:000664) mice, streptozotocin (STZ)-induced diabetic BL6 mice, C57BLKS/J (BLKS; IMSR Cat# JAX:000662, RRID:IMSR_JAX:000662) mice, C57BLKS/J-m+/+Lepr^db^ (db/+; IMSR Cat# JAX:000642, RRID:IMSR_JAX:000642) mice, C57BLKS/J-+Lepr^db^/+Lepr^db^ (db/db; IMSR Cat# JAX:000697, RRID:IMSR_JAX:000697) mice, and *Kir6.2*-deficient (*Kir6.2*^-/-^; IMSR Cat# RBRC09393, RRID:IMSR_RBRC09393) mice which were generated and backcrossed for more than five generations to BL6 background as described (Miki et al., 1998). Animals were housed in a temperature-controlled environment (22±2°C) on a 12-hour dark-light cycle with *ad libitum* access to food and water.

STZ-induced diabetic mice were acquired by intraperitoneal injection (i.p.) of 150 mg/kg STZ (Sigma-Aldrich Japan, Tokyo, Japan) in five-week-old male BL6 mice (*n* = 6) (Chubu Kagaku Shizai, Nagoya, Japan). Control non-diabetic mice (*n* = 6) received an equal volume of saline. Littermates of the same sex were randomly assigned to experimental groups. One week after STZ administration, the mice with plasma glucose concentrations of >16 mmol/l were selected as diabetic mice.

As it is known that db/db mice aged 24 weeks or older exhibit pathologically proven DPN (O'Brien et al., 2014), DRGs of STZ-treated BL6, BLKS, db/+, and db/db mice were harvested for qPCR at 24 weeks of age. To ensure that the db/db mice were obese and hyperglycemic, it was confirmed before sacrificing that the db/db mice had > 35 g of body weights and >16 mmol/l of random blood glucose levels. The Nagoya University Institutional Animal Care and Use Committee and the Aichi Medical University Institutional Animal Care and Use Committee approved the protocols of this experiment. The animal studies were carried out in accordance with the Act on Welfare and Management of Animals in Japan, the Animal Research: Reporting *In Vivo* Experiments (ARRIVE) guidelines, and the Act on the Conservation and Sustainable Use of Biological Diversity through Regulations on the Use of Living Modified Organisms.

#### DRG culture

DRG neuron cultures were prepared from DRGs collected from 5-week-old male BL6 mice (Chubu Kagaku Shizai), dissociated by collagenase (Wako Pure Chemical, Osaka, Japan) (n = 8-20 in each group), triturated through a series of heat-polished glass pipettes, and diluted in a medium consisting of Ham's F12 Nutrient Mixture (F12) media, 10 mmol/l glucose, and 30 nmol/l selenium. Isolated DRG neurons were seeded on glass coverslips coated with poly-L-lysine. The neurons were cultured with or without the following chemicals provided by Sigma Aldrich: glibenclamide (0.1 μmol/l, 1 μmol/l), tolbutamine (5 μmol/l, 50 μmol/l), and diazoxide (10 μmol/l, 100 μmol/l).

After 24 hours of culturing, DRG neurons fixed with 4% (wt/vol) PFA were immunostained with anti-Neurofilament H, clone NE14, Alexa Fluor 488 Conjugated antibody (1:5,000; Millipore Cat# MAB5256X, RRID: AB_11212967, Milford, MA). Neurite outgrowth was observed in 10 neurons per coverslip and evaluated by ImageJ software (National Institutes of Health). The total neurite length per neuron was measured using manual tracing. These experiments were repeated three times, and representative data are shown. Data acquisition and analyses were performed by personnel unaware of treatment assignment.

### Method details

#### Measurement of current perception threshold (CPT) using Neurometer^TM^

To determine a nociceptive threshold, CPT was measured in 4-, 12-, and 24-week *Kir6.2*^-/-^ and age-matched BL6 mice (*n* = 5-15 in each group) using a CPT/LAB Neurometer ^TM^ (Neurotron, Denver, CO). Each mouse was settled in a Ballman cage (Natsume Seisakusho, Tokyo, Japan) suitable for light restraint to keep awake. Two electrodes (SRE-0405-8; Neurotron) were attached to the plantar of one foot. Transcutaneous-sine wave electrical stimuli with three different frequencies (2000, 250, and 5 Hz) were applied to each plantar. The intensity of each stimulus gradually increased automatically (increments of 0.01 mA for 5 and 250 Hz, increments of 0.02 mA for 2000 Hz). The minimum intensity at which a mouse withdrew its paw was defined as the CPT. Six consecutive measurements were conducted at each frequency. Six consecutive measurements in each mouse were conducted at each frequency. Mice who were not settled in the cage were excluded from the experiment.

#### Thermal plantar test

Hind paw withdrawal response against thermal stimuli of radiant heat was measured at 12- and 24-week-old in Kir6.2^-/-^ and BL6 mice using radiant heat equipment (Plantar test model 7370; Ugo Basile, Comerio, Italy) according to the previously published method of Miura-Yura E, et al. (Himeno et al., 2013; Miura-Yura et al., 2020). In brief, the mouse put on a glass pane was stimulated by radiant heat placed below the glass pane. The hind paw withdrawal latencies were automatically recorded. The latencies were measured six times with a minimum interval of 5 minutes.

#### NCVs

Mice anesthetized with isoflurane were placed on a heated pad in a room maintained at 25°C to ensure a constant rectal temperature of 37°C. SNCVs were measured between the knee and ankle with orthodromic stimulation with Neuropak NEM-3102 instrument (Nihon-Koden, Osaka, Japan), as previously described (Himeno et al., 2015; Okawa et al., 2014). Paired needle electrodes were placed at the knee or ankle for stimulation and responses were recorded at the plantar.

#### Tissue collection

DRGs were harvested from each model of mice at 24 weeks old. Six lumbar DRGs obtained from each mouse were snap-frozen in liquid nitrogen followed by preservation at -80°C until use, and others were transferred to RNAlater Solution (Life Technologies Japan, Tokyo, Japan) followed by freezing preservation for qPCR. For immunohistochemistry, DRGs were fixed in 4% (wt/vol) paraformaldehyde (PFA) and frozen in O.C.T. compound (Sakura Finetek Japan, Tokyo, Japan) after cryo-protection with subsequent passages in increasing solutions of sucrose (10, 20, 30% (wt/vol)).

#### Real-time qPCR

Total RNA was extracted from DRGs using Qiagen RNAeasy kits (Qiagen, Valencia, CA) according to the manufacturer's instructions. Concentrations and purity of RNA were measured using a NanoDrop ND-1000 spectrophotometer (NanoDrop Technologies, Wilmington, DE). DNA was digested with RNase-free DNase I (Wako Pure Chemical, Osaka, Japan). DNase I was inactivated by incubation at 80°C for 10 min. Starting from 500 ng RNA in 50 μl of solution, cDNA was synthesized using ReverTraAce (Toyobo, Osaka, Japan), according to the manufacturer's instructions. Primers were designed by Primer3 software (http://frodo.wi.mit.edu/), spanned at least one intron, and confirmed the specificity using NCBI-BLAST (http://www.ncbi.nlm.nih.gov/tools/primer-blast/). Primer sequences are shown in Table S2. Real-time qPCR was performed and monitored using the StepOnePlus Real-Time PCR system (Applied Biosystems, Foster City, CA) with SYBR Green I as a double-stranded DNA-specific dye according to the manufacturer's instructions (Applied Biosystems). The PCR products were analyzed with a melting curve obtained at the end of each run and an agarose gel containing ethidium bromide to confirm these predicted lengths. Relative expression level was calculated using the delta-delta Ct method with normalization to beta-2 microglobulin.

#### Immunohistochemistry

For immunohistochemistry, after 5-minute microwave irradiation of cryostat sections in citrate buffer (pH 6.0), the sections were blocked with 5% (wt/vol) skim milk (Meiji, Tokyo, Japan) and the following primary antibodies were applied to the sections at 4°C overnight: rabbit polyclonal anti-KIR6.2 antibody ((1:200, Cat# 73-393, UC Davis/NIH NeuroMab Facility, Davis, CA, RRID: AB_2336908), anti-KIR6.1 antibody (1:200, Santa Cruz Biotechnology, Santa Cruz, CA, Cat# sc-11224, RRID: AB_2296514), anti-SUR1 antibody (1:200, Santa Cruz Biotechnology, Cat# sc-5789, RRID: AB_2219760), anti-SUR2A antibody (1:100, Santa Cruz Biotechnology, Cat# sc-32462, RRID: AB_2219765), and anti-SUR2B antibody (1:50, Santa Cruz Biotechnology Cat# sc-5793, RRID: AB_2219773). After washing with PBS, Alexa Fluor 488 or 594 conjugated anti-goat IgG antibodies (1:300, Thermo Fisher Scientific, Tokyo, Japan, Cat# A-11058, RRID: AB_2534105, or Cat# A-11055, RRID: AB_2534102) were loaded for 1 hour at room temperature. Coverslips and tissues were counterstained with DAPI (Merck, Tokyo, Japan). Images were captured by a CCD camera (DP70, Olympus Optical, Tokyo, Japan) using a fluorescence microscope (BX51, Olympus Optical).

#### Morphometry

*Kir6.2*^-/-^ and BL6 mice at 6 or 18 months old were sacrificed. They were anesthetized with a combination anesthetic, which was prepared with 0.3 mg/kg of medetomidine, 4.0 mg/kg of midazolam, and 5.0 mg/kg of butorphanol. Tissue was fixed by intracardiac perfusion with 1.6% PFA in PBS containing 1% glutaraldehyde. The sciatic and sural nerves were dissected and immersed in the same fixative for a further 1 hour at 4°C. The specimens were postfixed in 2% osmium tetroxide solution for 1 hour at 4°C, dehydrated in an ascending series of ethanol at room temperature, and embedded in Epon. Semithin sections (2 μm thick) were stained with toluidine blue to be viewed light microscopically for orientation. Ultrathin sections (80 nm thick) were cut on EMUC7i ultramicrotome (Leica Microsystems, Wetzlar, Germany) and stained with uranyl acetate and lead citrate. Images were captured using a transmission electron microscope JEM-1400 (JEOL, Tokyo, Japan) and analyzed using ImageJ software (National Institutes of Health, Bethesda, MD).

For counting numbers of myelinated fibers, systematically selected frames representing 40 to 80% of the sural nerve cross-sectional area were photographed. For unmyelinated fibers, 20 to 40% of the area was photographed. The total numbers of myelinated or unmyelinated fibers were calculated based on the size of the sural nerve endoneurial area measured from semithin sections. The *g*-ratio, the ratio of the inner axonal diameter to the total outer diameter was measured using AxonSeg software (https://github.com/neuropoly/axonseg).

### Quantification and statistical analysis

All the group values were expressed as mean±SD. Data of *ex vivo* experiments are representative of at least three independent experiments. The normality of distribution was tested by the Kolmogorov-Smirnov test using R version 3.4.3 (http://www.r-project.org/, Vienna, Austria). Statistical analyses were conducted by unpaired Student's t-test (comparisons of mRNA expression levels of SUR1, SUR2B, Kir6.1, and Kir6.2 between non-diabetic and diabetic BL6; comparisons of NCVs, CPTs, and thermal perception thresholds in each age between BL6 and *Kir6.2*^-/-^ mice; comparisons of nerve fiber number and size between BL6 and *Kir6.2*^-/-^) or one-way ANOVA with the Bonferroni correction for multiple comparisons (comparisons of mRNA expression levels of SUR1, SUR2B, Kir6.1, and Kir6.2 among BLKS, db/+, and db/db mice; comparisons of neurite lengths among different concentrations of K_ATP_ channel closers and openers) using StatView version 5.0 (SAS Institute, Cary, NC), as previously described (Mohiuddin et al., 2019). The threshold of statistical significance was taken as a value of p < 0.05. The values of *n* represent numbers of mice in Figures 1, 2, and Figure 4, Figure 5, Figure 6, Figure 7 and numbers of wells in Figure 3. The current experiments did not need randomization of mice. All analyses were performed by personnel unaware of the animal identities.

### Additional resources

Not applicable.

## Data Availability

All data reported in this paper will be shared by the lead contact upon request. This paper does not report original code. Any additional information required to reanalyze the data reported in this paper is available from the lead contact upon request.
