# Supplementary material for: Kir6.2-deficient mice develop somatosensory dysfunction and axonal loss in the peripheral nerves
Source: iScience. 2021 Dec 11;25(1):103609. doi: 10.1016/j.isci.2021.103609 (PMC8719014; doi:10.1016/j.isci.2021.103609)

iScience, Volume *25*

Supplemental information

Kir6.2-deficient mice develop somatosensory dysfunction and axonal loss in the peripheral nerves

Hiromi Nakai-Shimoda, Tatsuhito Himeno, Tetsuji Okawa, Emiri Miura-Yura, Sachiko Sasajima, Makoto Kato, Yuichiro Yamada, Yoshiaki Morishita, Shin Tsunekawa, Yoshiro Kato, Yusuke Seino, Rieko Inoue, Masaki Kondo, Susumu Seino, Keiko Naruse, Koichi Kato, Hiroki Mizukami, Jiro Nakamura, and Hideki Kamiya

Table S1. Quantification of morphometry in the sciatic nerve using electron microscopy, Related to Table 1

	C57BL6/J		*Kir6.2*$^{-/-}$		*p*
	mean	SD	mean	SD	
Myelinated fiber size (µm^2)	26.8	6.7	25.8	4.1	0.856
Mean myelin area (µm^2)	13.3	2.9	13.4	0.9	0.951
Mean myelin thickness (µm)	0.82	0.07	0.87	0.05	0.387
Mean axon area (µm^2)	13.6	3.9	12.4	3.3	0.753
g-ratio	0.67	0.01	0.64	0.03	0.305

Table S2. Sequences of qPCR primers used in this study, Related to STAR methods

Gene abbreviation	Full name	Forward primer (5'-3')	Reverse primer (3'-5')	Intron spanning	NCBI Gene ID
Kcnj8	Potassium inwardly-rectifying channel, subfamily J, member 8	CTATCATGTGGTGGCTGGTG	CGTGAATGACCTGACATTGG	yes	16523
Kcnj11	Potassium inwardly rectifying channel, subfamily J, member 11	CAAGATGCACTTCAGGCAAA	CCAGGCTGAACTTCCCAATA	yes	16514
Abcc8	ATP-binding cassette, sub-family C (CFTR/MRP), member 8	CAAGGTGTCCTCAACAACGG	CCAGGTGCTATGGTGAATGTG	yes	20927
Abcc9	ATP-binding cassette, sub-family C (CFTR/MRP), member 9	ATGAAGCCACTGCTTCCATC (for *SUR2A* transcript variant) CCATAGCTCATCGGGTTCAC (for *SUR2B* transcript variant)	AAGGCCTGCATCCACAATAG (for *SUR2A* transcript variant) AACGAGGCAAACACTCCATC (for *SUR2B* transcript variant)	yes	20928
B2m	Beta-2 microglobulin	TTCTGGTGCTTGTCTCACTGA	CAGTATGTTCGGCTTCCCATTC	yes	12010

NCBI: National Center for Biotechnology Information

Figure S1. Real-time qPCR of K_{ATP} channels in dorsal root ganglia (DRG) of wild-type or obese diabetic mice. Related to Figure 1.

Expression levels of K_{ATP} channel component proteins in DRG of C57BLKS (BLKS), db/+, or db/db fatty diabetic mice. *Sur1*, *Sur2B*, *Kcnj11*, and *Kcnj8* were expressed in the DRG of all three types of mice. The expression levels of *Sur2B* and *Kcnj8* in db/db mice significantly increased compared with those in BLKS mice. The levels of *Sur1* in db/+ mice increased compared with those in BLKS mice. SUR: sulfonylurea receptor, Kir: inward rectifier potassium channel, BLKS: C57BLKS mouse, db/+: C57BLKS/J-m+/+Leprdb (db/+) mouse, db/db: C57BLKS/J-+$Lepr^{db}$/+$Lepr^{db}$ (db/db) mouse. *: p<0.01 versus BLKS, **: p<0.001 versus BLKS, †: p<0.01 vs db/+.

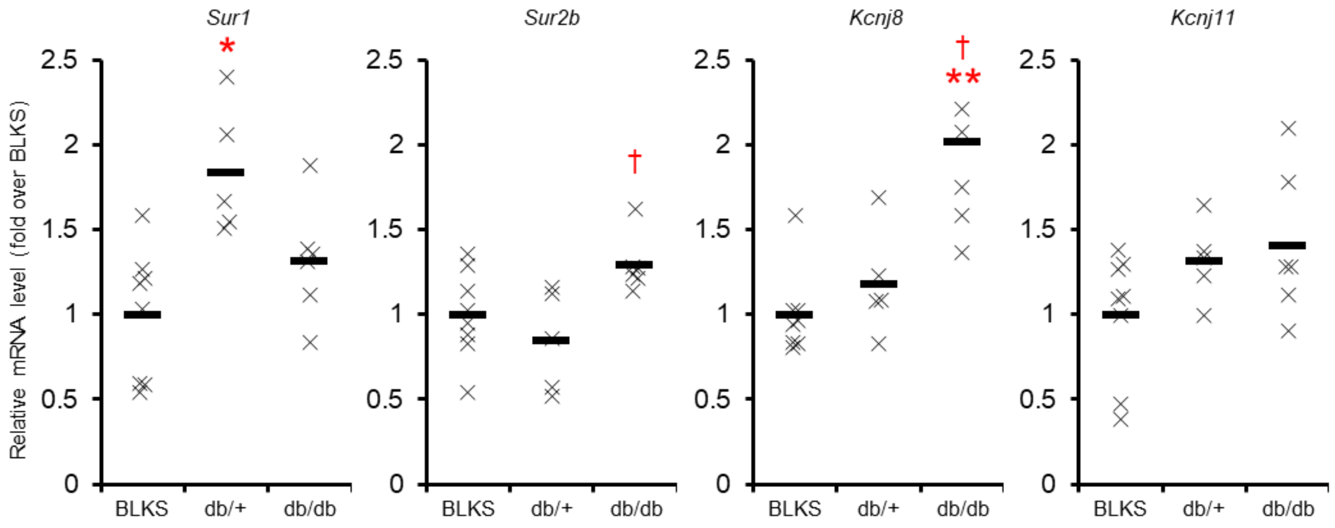

Figure S2. An electric microphotograph of the sural nerve in *Kir6.2*-deficient mouse. Related to Figure 6.

There are three phenotypes of Remak bundles; an intact bundle filled by axons and Schwann cell membranes without cytoplasm (arrow), bundles with empty gaps which can be inflated Schwann cell tongues (*), and a denervated Schwann cell profile (arrowhead)

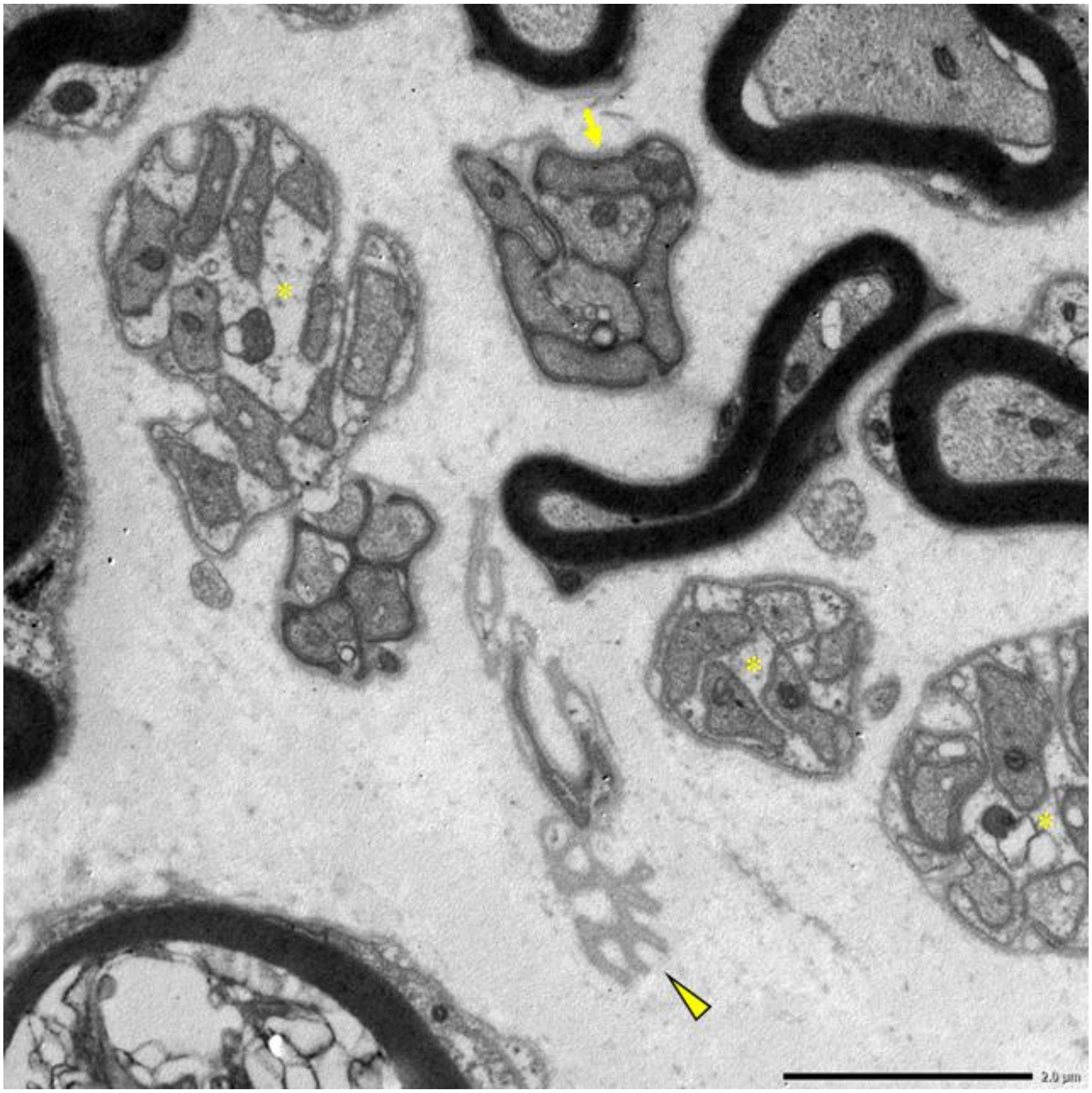

Figure S3. This photograph displays the heterogeneity of nerve involvement in *Kir6.2*$^{-/-}$ mice. Related to Figure 6.

The left side of the Remak bundle indicates degeneration of unmyelinated axons but the right side, which is held by another Schwann cell tongues, appears to be intact. Arrows indicate edges of Schwann cell tongues.

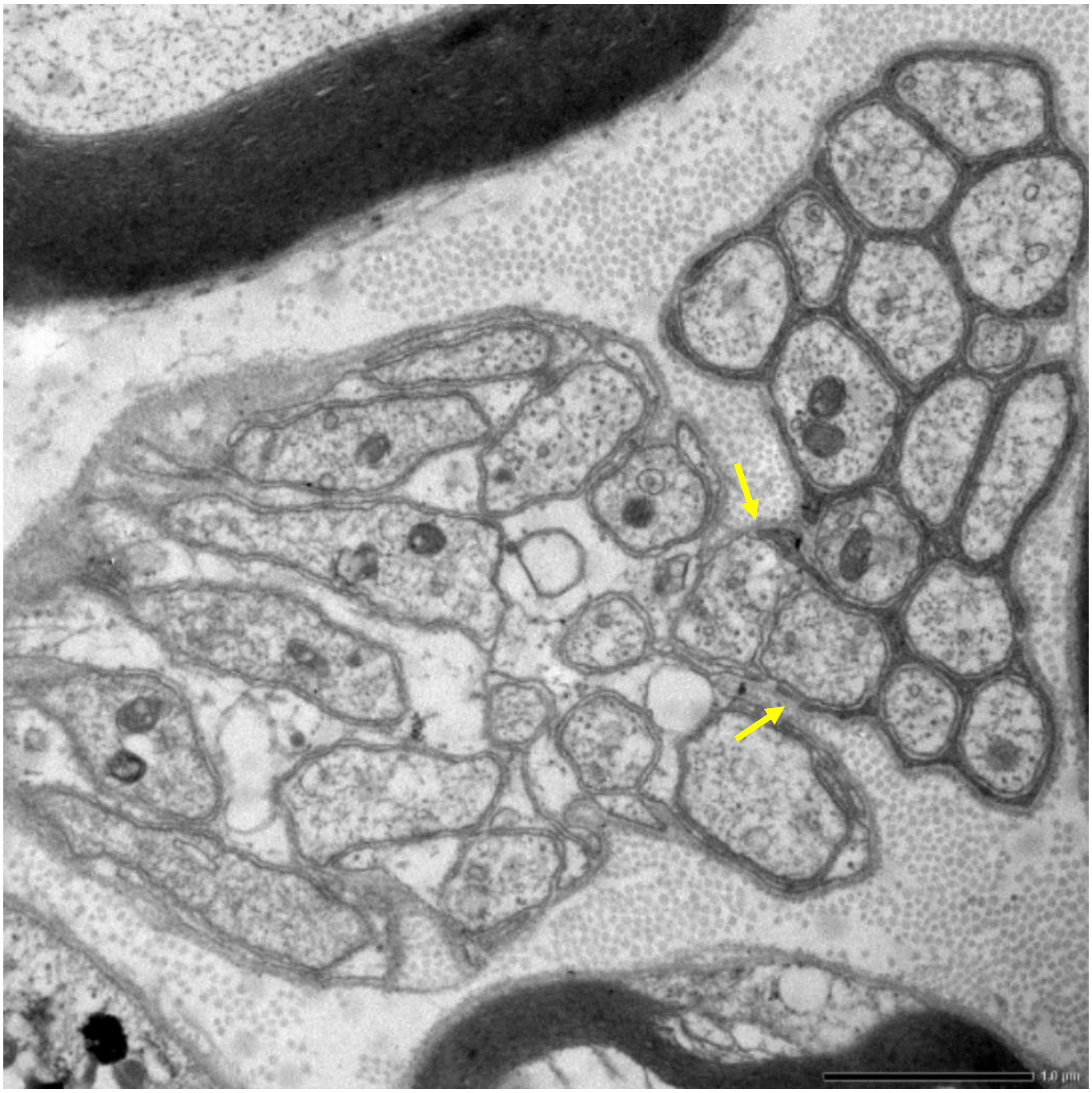

Figure S4. Histograms of myelinated fibers in the sural nerve. Related to Figure 7.

A: A frequency histogram of axon area. B: A histogram of estimated numbers of axons with each size in the whole sural nerve. C: A frequency histogram of myelin area. Open bars are C57BL6/J mice. Solid bars are $\mathit{Kir6.2}^{-/-}$ mice. n = 5 in $\mathit{Kir6.2}^{-/-}$ mice, n = 4 in C57BL6/J mice. *: $p < 0.05$ compared with C57BL6/J mice. Data are represented as mean±SD.

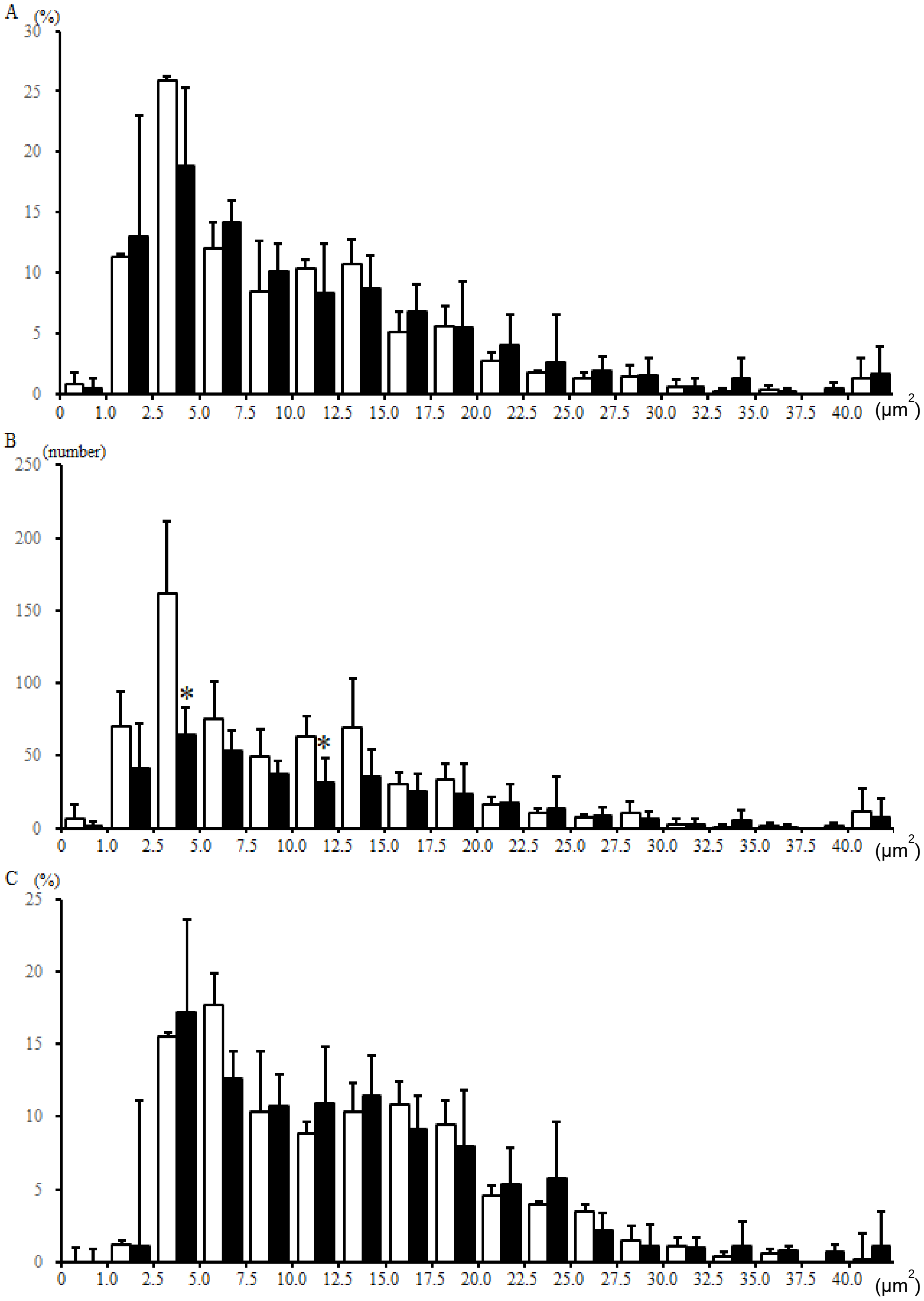

Figure S5. An electric microphotograph of the sciatic nerve in *Kir6.2*-deficient mouse. Related to Figure 7.

Onion bulb-like structure with thin myelin sheath (*) and clusters of regenerating myelinated axons(*ra*) are seen. Scale bar: 5 μm.

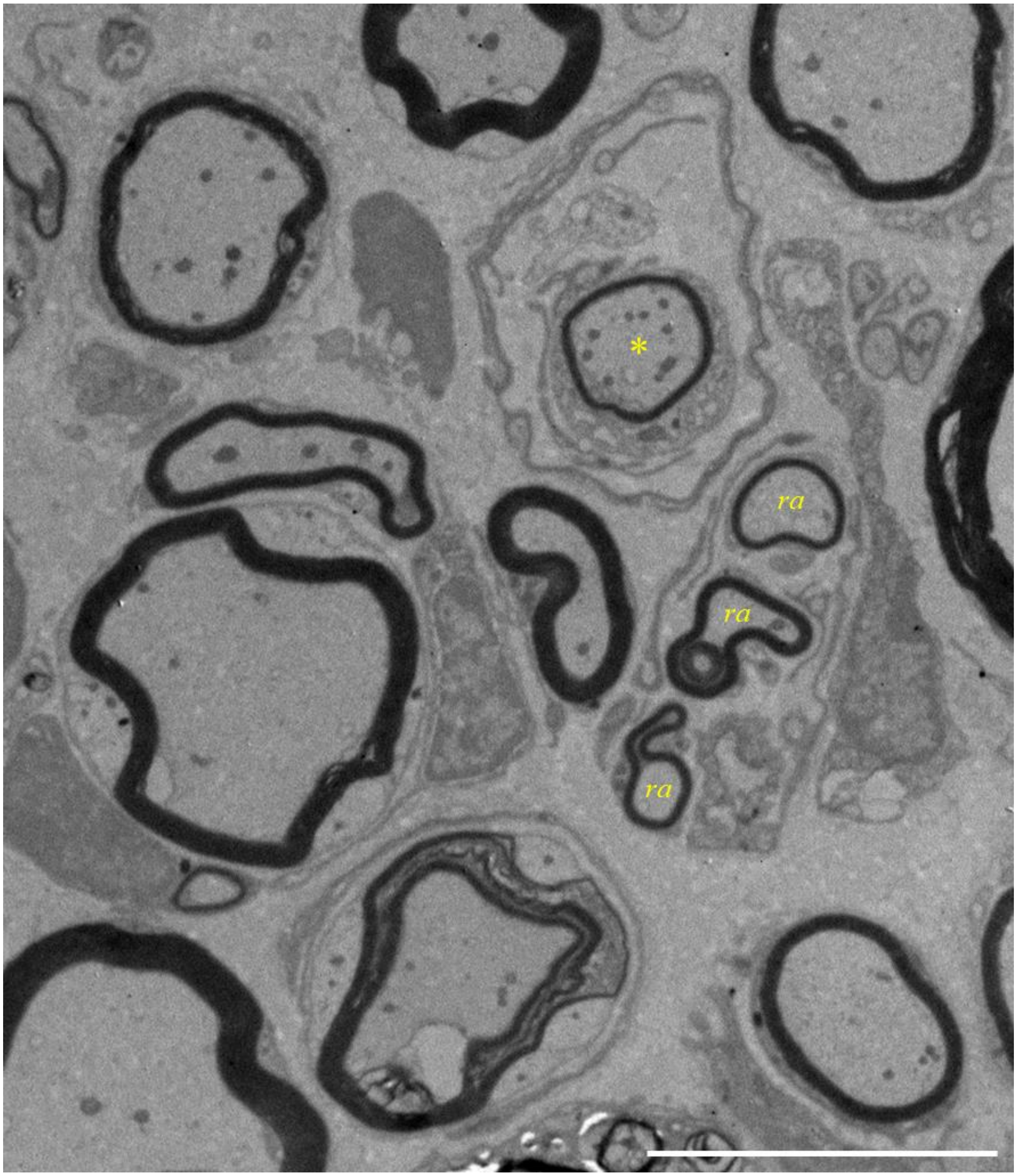

Supplement: Document S1. Figures S1–S5 and Tables S1 — and S2 [file mmc1.pdf]
